# Study protocol of an observational study in acute psychiatric home treatment: How does home treatment work? Identification of common factors and predictors of treatment success

**DOI:** 10.1007/s40211-023-00457-0

**Published:** 2023-03-20

**Authors:** Felix Baumann, Vera Bergamaschi, Ingeborg Warnke, Salvatore Corbisiero, Kerstin Gabriel Felleiter, Seraina Fellmann, Fabian Ludwig, Andreas Riedel, Hansjörg Znoj, Stefanie Schmidt

**Affiliations:** 1 Institute of Psychology, University of Berne, Berne, Switzerland; 2 Lucerne Psychiatry, Lucerne, Switzerland; 3 GiA Stadt, Lucerne Psychiatry, Voltastr. 42, 6005 Lucerne, Switzerland

**Keywords:** Mental health services, Acute psychiatry, Therapeutic relationship, Social aspects, Follow-up, Psychiatrische Behandlung, Akutpsychiatrie, Therapiebeziehung, Soziale Aspekte, Follow-up

## Abstract

**Background:**

Systematic reviews indicated that home treatment is an effective and cost-saving alternative to conventional acute psychiatric treatment options. Treatment success has often been defined as a reduction of hospital admissions. In the current study, symptoms and well-being are assessed regularly during treatment as an indicator for treatment success. Patients’ characteristics such as diagnosis, age, substance use, and motivation for treatment were discussed as predictors for treatment success. A second focal point of the study lies in the examination of the therapeutic relationship in terms of the outcome, which has not yet been systematically investigated in home treatment.

**Methods:**

This is an observational study with a prospective naturalistic design. Measurements are carried out at baseline, during and at the end of treatment as well as at the 3‑month follow-up. Patients’ characteristics as potential predictors for treatment success will be assessed at baseline. In addition, the perceived relationship between the patients and the team will be measured daily and weekly throughout the treatment. Treatment success is by the changes in symptoms and general well-being assessed weekly. We aim to include 82 participants assigned to home treatment. Variance analyses with repeated measurements will be conducted to evaluate treatment success.

**Conclusion:**

By examining potential patient- and relationship-related predictors of treatment success, insights into relevant determining variables of treatment success in this setting are expected. The results might help to better identify who benefits the most from home treatment.

## Abstract (de)

**Hintergrund:**

Systematische Übersichtsarbeiten haben ergeben, dass *Home Treatment* eine wirksame und kostensparende Alternative zu herkömmlichen akutpsychiatrischen Behandlungsmöglichkeiten darstellt. Der Behandlungserfolg wird häufig über eine Reduktion der Klinikeintritte definiert. In der aktuellen Studie werden Symptomatik und Wohlbefinden regelmäßig als Indikator für den Behandlungserfolg erhoben. Charakteristika wie Diagnose, Alter, Substanzkonsum und Behandlungsmotivation wurden als Prädiktoren für den Behandlungserfolg diskutiert. Der zweite Schwerpunkt liegt in der Untersuchung der Therapiebeziehung in Bezug auf die Wirksamkeit, welche im Home Treatment bisher noch nicht systematisch untersucht wurde.

**Methode:**

Es handelt sich um eine Beobachtungsstudie mit einem prospektiven naturalistischen Design. Die Messungen werden vor, während und nach der Behandlung mit einem dreimonatigen Follow-up durchgeführt. Charakteristika der Patientinnen und Patienten werden zu Beginn der Behandlung als mögliche Prädiktoren des Behandlungserfolgs erhoben. Darüber hinaus wird die Therapiebeziehung zwischen den Patientinnen/Patienten und dem Team während der Behandlung regelmäßig gemessen. Der Behandlungserfolg wird anhand der Veränderungen der Symptome und des allgemeinen Wohlbefindens definiert, die jeweils wöchentlich gemessen werden. Es wird angestrebt, 82 Teilnehmer in das Home Treatment einzubeziehen. Zur Bewertung des Behandlungserfolgs werden Varianzanalysen mit Messwiederholung durchgeführt.

**Schlussfolgerung:**

Durch die Analyse von Prädiktoren für den Behandlungserfolg auf Patienten- und Beziehungsebene werden Einblicke zu wichtigen Determinanten für den Behandlungserfolg im Home Treatment erwartet. Die Ergebnisse tragen zum Verständnis bei, wer von Home Treatment profitieren kann.

## Background

Home treatment (HT) offers an intensive acute psychiatric treatment in the patients’ domestic environment as an alternative to inpatient care for over 40 years [1]. HT aims to replace inpatient treatment for patients in an acute psychiatric crisis [2]. Daily contacts are provided through a mobile and interdisciplinary team for a limited treatment period. To ensure safety at home, a 24 h emergency phone can be provided. Systematic reviews indicated that HT is a feasible and effective alternative to conventional inpatient treatment [3, 4]. Nowadays, HT options have been implemented in several, foremost western countries [5–9].

Recent studies could show the success of HT in Switzerland. Significant increases in clinical and social functioning were found for patients with severe and acute mental illness from admission to discharge [9, 10]. Most of these studies on HT used either the criterion of admission to inpatient treatment [11, 12] or changes in clinical routine data [7, 8] to define success of HT. Only few studies focused on more specific or clinical outcome measures, e.g., self-efficacy and changes in symptoms [6, 9]. A so far neglected concept in the field of HT is emotion regulation. In a systematic review [13], it was found that regardless of the intervention or disorder, maladaptive emotion regulation decreased post/during treatment.

Few studies in the field of acute psychiatric HT aimed to identify patient-level factors related to the success of this form of treatment. Being older and living in socially disadvantaged areas [14, 15], previous hospital stays [16], and psychotic disorders [15] predicted a worse treatment outcome (i.e., relapse after HT). However, symptomatology and age could not consistently be confirmed as significant predictors for treatment success [16]. Overall, it can be stated that existing research on predictors for treatment success is rather inconsistent, and findings were often a by-product of studies designed to investigate the overall effectiveness of HT.

Besides the predictors on a patient-level mentioned above, qualitative research indicates that the therapeutic relationship is an important factor in HT [17]. The therapeutic relationship describes the interpersonal contact between patients and therapists [18]. It acts independently of disorder-specific interventions and is therefore known as a common factor [19]. Empirical evidence consistently shows a moderate positive correlation between the therapeutic relationship and treatment outcome [20, 21].

The treatment setting itself appears to have an influence on the development and establishment of this relationship. A systematic review demonstrated that building and maintaining a good therapeutic relationship could be difficult in an acute psychiatric setting compared to scheduled, long-term therapeutic sessions due to several factors, i.e., the setting itself, patient attributes and staff attributes [22]. Outpatients could establish a stronger relationship than inpatients because of the decreased severity of psychopathology [23] and a lower number of involved specialists [24]. Presumably, the collaboration of an entire treatment team diminishes the influence of a single therapist on the patient’s treatment outcome [25]. Qualitative research has shown that the therapeutic relationship is a crucial factor in achieving therapeutic success in a HT setting [17], whereby there are still hardly any findings regarding the importance of the therapeutic relationship in HT for acute psychiatric patients.

The present study uses an explorative prospective naturalistic design with multiple measurements of symptoms and well-being during treatment to dynamically assess the therapeutic process and outcome of HT. The study has two main research questions: identification of patient-level predictors for treatment success in HT and modelling the course of the therapeutic relationship in this setting. According to previous findings we hypothesize that age, gender, diagnosis, number of previous hospitalizations, social living situation as well as the therapeutic relationship during treatment serve as predictors for the treatment success of HT.

## Methods

### Setting

The study takes place at the HT service in Lucerne and agglomeration (approximately 235,000 residents) offered by Lucerne Psychiatry since 2007. In 2020, 234 patients in the whole psychiatric spectrum were treated in their domestic environment. In average, treatment lasted for 41 days. At least once a day, patients are visited by one member of the interdisciplinary treatment team, which consists of 12 psychiatric nurses, 3 psychologists, and 4 medical doctors. At the beginning, for each patient a core team, consisting of 3 specialists from the respective professional groups, draws up an individual treatment plan together with the patient. Many of the daily visits are handled by a member of the core team. However, because the entire team works in shifts, patients are also visited by other team members. Through daily rapport, all team members are involved in the treatment ensuring treatment consistency.

Patients can participate in several group therapies at the inpatient hospital of Lucerne Psychiatry. A 24 h emergency service is provided by the team. In case of a psychiatric emergency during HT, a bed in the inpatient hospital of Lucerne Psychiatry is provided. Patients can stay there for up to 7 days with daily visits by the HT team. If inpatient care is needed for more than 1 week, patients are fully admitted to hospital. During HT, patients get a sickness certification with the possibility of starting a therapeutic work trial in their regular employment or in a protected work environment. Patients are supported in the search and initiation of a suitable follow-up treatment, e.g., outpatient psychotherapy.

### Sample

The sample consists of patients assigned to the acute psychiatric HT in Lucerne. They are referred to HT by institutions and clinical practitioners. Minimal age for treatment is 18 years and patients with all psychiatric diagnoses are treated. As a prerequisite for treatment, patients must be able to distance themselves from acute danger to themselves or others. Furthermore, patients fulfilling the following inclusion criteria are eligible for the study: patients admitted to the examined HT, agree to participate voluntarily. Informed consent is given as documented by signature. Excluded are patients unable to follow the procedures of the study due to language problems. Study participation does not influence the treatment and participation is not compensated.

### Procedures

Patients are assigned to the acute psychiatric HT. Once the referral is received, the project leaders send a flyer and template of informed consent by mail to the assigned patients to inform them about the study. The mail is followed by a phone call to personally inform them about the study. Patients can be included in the study, if their verbal and written consent is obtained. Patients have time to decide whether to participate or not (at least 24 h before). Time to start of treatment varies depending on current capacity. In 2019 the average time to start of treatment was 8 days.

**Fig. 1:**
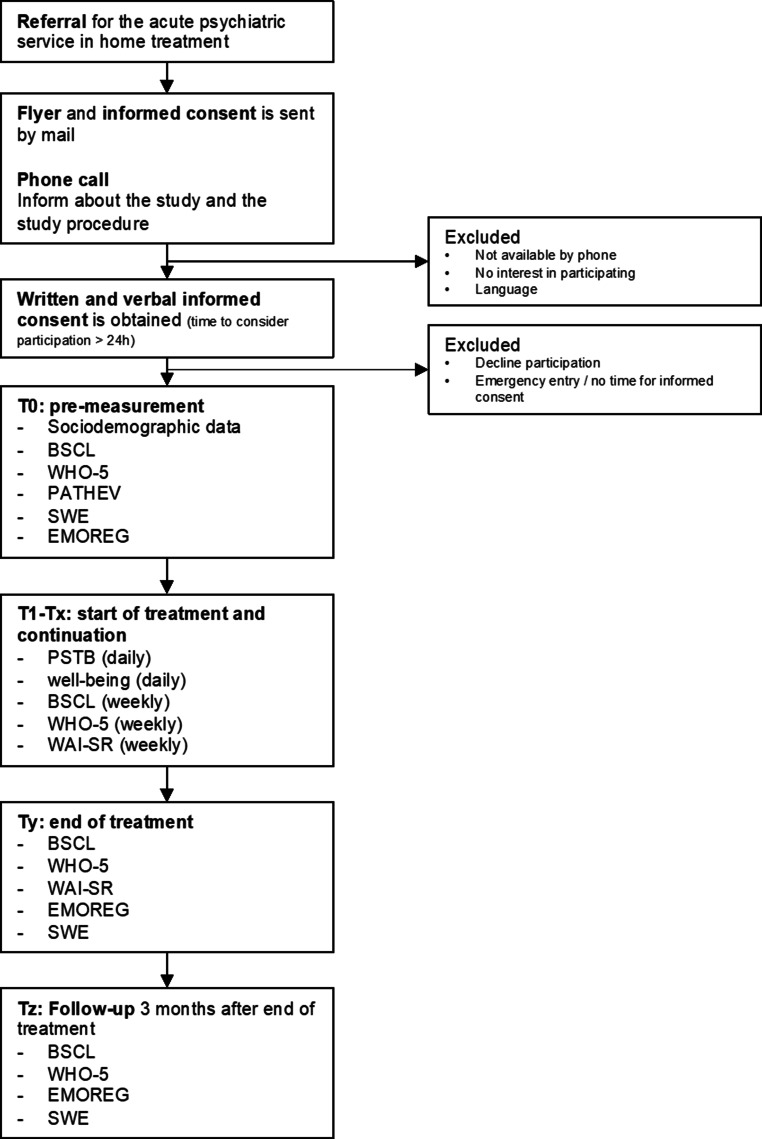
Flowchart of study procedures. *BSCL* Brief Symptom Checklist, *WHO‑5* World Health Organization–Five Well-Being Index, *PATHEV* Patient Questionnaire on Therapy Expectation and Evaluation, *SWE* General Perceived Self-Efficacy Scale, *EMOREG* Fragebogen zur Emotionsregulation, *PSTB* The Bern Post Session Report, *WAI-SR* Working Alliance Inventory—Short Revised, *Well-being* self-constructed measurement on a rating scale from 1 (very bad) to 10 (very well) based on the common question in psychiatric practice about the current state of well-being

In case of participation, the link to the first set of questionnaires is sent by mail (paper versions are available if requested), which they fill out until the start of treatment (Fig. 1).

The study protocol and procedures were approved by the Swiss ethics committee Nordwest- und Zentralschweiz (EKZN: 2020-02736).

### Measurements

The first set of questionnaires aims to assess patient’ characteristics as potential predictors for treatment outcome (focal point of first research question). The following sociodemographic data are assessed: age, gender, social living situation, relationship status, highest degree, job situation before treatment, previous inpatient hospitalization, and previous HT. Symptoms are measured with the German version of Brief Symptom Checklist (BSCL) [26]. BSCL consists of 53 items rated on a 5-point Likert scale. Well-being is assessed with the five items of the German version of World Health Organization–Five Well-Being Index (WHO-5) on a 6-point Likert scale [27]. Therapy expectation and evaluation are measured with the German version of Patient Questionnaire on Therapy Expectation and Evaluation (PATHEV) [28], which consists of 11 items rated on a 5-point Likert scale. Ten items of self-efficacy are assessed with the German version of General Perceived Self-Efficacy Scale (SWE) [29] on a 4-point Likert scale and emotion regulation is measured with the “Fragebogen zur Emotionsregulation” (EMOREG) [30] with 26 items on a 6-point Likert scale. Clinical diagnosis is also included. It is carried out as part of the clinical assessment and subsequent treatment by specialists (psychiatrists, psychologists).

For the second focal point, therapeutic relationship is assessed with three items on a 7-point Likert scale of the German version of Bern Post Session Report (PSTB) [31]. Well-being is measured with a self-constructed daily measurement on a numeric rating scale from 1 (very bad) to 10 (very well) based on the common question in psychiatric practice about the current state of well-being. Both questionnaires are assessed daily. The profession and function (e.g., member of the core team) of the visiting team member as well as the type of the treatment (amount of emergency calls, participation in group therapy) for each participant visit is documented separately.

Working alliance is measured weekly with the German version of Working Alliance Inventory—Short Revised (WAI-SR) [24]. WAI-SR includes 12 items rated on a 5-point Likert scale.

For both research questions, BSCL and WHO‑5 are measured weekly throughout, at the end of treatment and 3 months following treatment. Changes in their scores over time represent the primary outcome as a measure of treatment success.

At the end of treatment patients fill out WAI-SR, EMOREG and SWE in addition to BSCL and WHO‑5. Three months after the end of the treatment, the project leaders contact the participants by phone. They receive a last set of questionnaires (BSCL, WHO‑5, EMOREG and SWE; Table 1 for the schedule of assessments).

**Table 1:** Schedule of assessments

Instrument	Domain	Baseline	Treatment daily	Treatment weekly	End of treatment	Follow-up 3 months
Sociodemographic data	–	X	–	–	–	–
PATHEV	Therapy expectation and evaluation	X	–	–	–	–
SWE	Self-efficacy	X	–	–	X	X
EMOREG	Emotional regulation	X	–	–	X	X
PSTB	Working alliance	–	X	–	–	–
Well-being	1–10 (own scale)	–	X	–	–	–
WAI-SR	Working alliance	–	–	X	X	–
BSCL	Symptom level	X	–	X	X	X
WHO‑5	Well-being	X	–	X	X	X

*PATHEV* Patient Questionnaire on Therapy Expectation and Evaluation, *SWE* General Perceived Self-Efficacy Scale, *EMOREG* Fragebogen zur Emotionsregulation, *PSTB* The Bern Post Session Report, *WAI-SR* Working Alliance Inventory—short revised, *BSCL* Brief-Symptom-Checklist, *WHO‑5* Well-Being Index

### Statistical analysis

#### Definition of the sample size

A power-analysis was carried out to determine the sample size. We calculated with a F-test for analysis of variance (ANOVA), repeated measures, within factors, one group and three measurement-points (baseline, post and follow-up). The power analysis (G * Power 3.1.9.4) [32] showed a sample size of 43 for an effect size of *f* = 0.25 with an α error of 0.05 and actual power of 0.95. The effect size was chosen based on findings in psychiatric research [33].

In 2019, around 30% of the registered patients did not start treatment. In addition, the treatment duration was less than a week in 12% of the treatments. Finally, a discontinuation rate of 30% was anticipated for the follow-up according to previous literature [34, 35]. To compensate for these foreseeable losses, the final sample size was adjusted to at least 82. Drop-outs will be analyzed. Random missing values will be supplemented with the “multiple imputation” method, categorical missing values are supplemented by last observation forward as indicated.

#### Statistical models

A general linear mixed model (GLMM) [36] was initially planned. Experience with recruitment indicates that the planned sample size is unlikely to be achieved, which has implications for statistical model. Therefore, treatment success as main outcome is evaluated with a repeated measures analysis of variance (ANOVA [37]) model. Data will be analyzed by SPSS Statistics (version 26, IBM, Armonk, NY, USA) [38].

Treatment success is defined by the comparison of baseline and post measurements of symptoms (BSCL) and well-being (WHO-5) as dependent variables. Further, regression analysis is carried out to assess the predictive value of patient level characteristics (baseline level of BSCL and WHO‑5, SWE, EMOREG, sociodemographic data, diagnosis and PATHEV) as independent variables for treatment success as dependent variable.

Concerning the therapeutic relationship, two separate linear regression analyses will be carried out: one based on weekly measurements (WAI-SR) and one based on daily measurements (PSTB). The therapeutic relationship functions as the independent variable in both analyses. Treatment success as defined above and daily well-being act as dependent variables.

To explore the assumption of an association between the relationship to a treatment team in general (WAI-SR) and the relationship to the individual members of the team on each visit (PSTB), a linear regression analysis is carried out with the WAI-SR as a dependent and the PTSB as independent variable.

## Discussion

The aim of this study is to demonstrate that HT leads to significant changes in symptoms and well-being. This could create a strong argument for a national implementation of HT. Similar treatment options are currently being developed in many places in Switzerland.

Exploratory findings on the importance of the therapeutic relationship in HT might provide recommendations for improving structures and the organization of an interdisciplinary treatment team. Previous research indicates that patients in HT experience the relationship with the treatment team on an equal footing, which made it challenging for the treatment team to maintain professionalism and boundaries [21].

Researching the predictive value of patient characteristics for treatment outcome, including follow-up, can provide initial indications of who can benefit from this setting. Variables such as emotionally unstable personality disorder, self-harming behavior, substance abuse and previous psychiatric admissions [39] as well as schizophrenia, psychotic disorders, severe mood disorders, personality disorders, low socioeconomic status, living alone, employment status, sick/disability pay, and previous psychiatric treatments [40] were associated with repeated use of psychiatric treatments.

Predictor analysis may help to specify the indication for HT based on certain patient characteristics as well as the adaptation of the treatment offer to different patient groups.

The lack of a control group and the size of the planned sample are limiting factors for the validity and generalizability of the study. The design was chosen to ensure the feasibility of the study in the context of existing and ongoing treatment.
